# Amphetamine Induces Oxidative Stress, Glial Activation and Transient Angiogenesis in Prefrontal Cortex via AT_1_-R

**DOI:** 10.3389/fphar.2021.647747

**Published:** 2021-05-03

**Authors:** Osvaldo M. Basmadjian, Victoria B. Occhieppo, Natalia A. Marchese, M. Jazmin Silvero C., María Cecilia Becerra, Gustavo Baiardi, Claudia Bregonzio

**Affiliations:** ^1^ Departamento de Farmacología, Facultad de Ciencias Químicas, Instituto de Farmacología Experimental Córdoba (IFEC-CONICET), Universidad Nacional de Córdoba, Córdoba, Argentina; ^2^ Centro de Investigaciones en Química Biológica de Córdoba (CIQUIBIC), CONICET, Facultad de Ciencias Químicas, Universidad Nacional de Córdoba, Córdoba, Argentina; ^3^ Departamento de Química Biológica “Ranwel Caputto”, Facultad de Ciencias Químicas, Universidad Nacional de Córdoba, Córdoba, Argentina; ^4^ Instituto Multidisciplinario de Biología Vegetal (IMBIV-CONICET) Departamento de Ciencias Farmacéuticas, Facultad de Ciencias Químicas, Universidad Nacional de Córdoba, Córdoba, Argentina; ^5^ Laboratorio de Neurofarmacología, (IIBYT-CONICET), Universidad Nacional de Córdoba, Córdoba, Argentina; ^6^ Facultad de Ciencias Químicas, Universidad Católica de Córdoba, Córdoba, Argentina

**Keywords:** AT1 receptor, oxidative stress, angiogeneis, amphetamine, glia, working memory, short-term memory, prefrontal cortex

## Abstract

**Background:** Amphetamine (AMPH) alters neurons, glia and microvessels, which affects neurovascular unit coupling, leading to disruption in brain functions such as attention and working memory. Oxidative stress plays a crucial role in these alterations. The angiotensin type I receptors (AT_1_-R) mediate deleterious effects, such as oxidative/inflammatory responses, endothelial dysfunction, neuronal oxidative damage, alterations that overlap with those observed from AMPH exposure.

**Aims:** The aim of this study was to evaluate the AT_1_-R role in AMPH-induced oxidative stress and glial and vascular alterations in the prefrontal cortex (PFC). Furthermore, we aimed to evaluate the involvement of AT_1_-R in the AMPH-induced short-term memory and working memory deficit.

**Methods:** Male Wistar rats were repeatedly administered with the AT_1_-R blocker candesartan (CAND) and AMPH. Acute oxidative stress in the PFC was evaluated immediately after the last AMPH administration by determining lipid and protein peroxidation. After 21 off-drug days, long-lasting alterations in the glia, microvessel architecture and to cognitive tasks were evaluated by GFAP, CD11b and von Willebrand immunostaining and by short-term and working memory assessment.

**Results:** AMPH induced acute oxidative stress, long-lasting glial reactivity in the PFC and a working memory deficit that were prevented by AT_1_-R blockade pretreatment. Moreover, AMPH induces transient angiogenesis in PFC via AT_1_-R. AMPH did not affect short-term memory.

**Conclusion:** Our results support the protective role of AT_1_-R blockade in AMPH-induced oxidative stress, transient angiogenesis and long-lasting glial activation, preserving working memory performance.

## Introduction

The psychostimulant properties of amphetamine (AMPH) make it useful for the treatment of some psychiatric diseases such as attention deficit hyperactivity disorder and narcolepsy; however, it has a high potential for abuse. AMPH and methamphetamine, after opioids, are the major contributors to the global disease burden attributable to drug use disorders (United Nations, 2017). In addition to its acute psychostimulant effects, AMPH exposure is also associated with a broad range of long-lasting changes in all components of the neurovascular unit. Neuronal, glial and vessel functioning are altered, losing suitable coupling between neuronal activity and nutrient delivery (Kalivas, 2007; Carvalho et al., 2012; Paz et al., 2013; Paz et al., 2014; Occhieppo et al., 2017; Schrantee et al., 2017).

Oxidative stress plays a crucial role in AMPH-induced neurotoxicity and glial activation (Moratalla et al., 2017). Thus, AMPH-induced mitochondrial dysfunction and dopamine oxidation lead to increased production of reactive oxygen species (ROS) (Moratalla et al., 2017; Tung et al., 2017). Reactive nitrogen species production is also induced by the increase of Ca^2+^ influx and oxide nitric synthase activity (Brown and Yamamoto, 2003). In consequence, these reactive compounds oxidize and alter the functioning of several cellular elements, including lipids, proteins and nucleic acids. AMPH-induced oxidative damage may subsequently induce microglial activation (Shaerzadeh et al., 2018). The excessive activation of these cells appears to contribute to some neurotoxic effects of AMPH, such as dopamine nerve endings degeneration (LaVoie et al., 2004). Microglial activation is also typically followed by astroglial activation, which contributes to increased levels of proinflammatory cytokines (Loftis and Janoswsky, 2014; Shaerzadeh et al., 2018). In rodents, amphetamines-induced glial reactivity and neuroinflammation are usually observed in brain areas that have dopaminergic endings such as striatum, hippocampus, thalamus and parietal cortex (Shaerzadeh et al., 2018). Interestingly, microglial hypertrophy is pronounced in the mice hippocampus exposed to amphetamines, while little changes are observed in this brain area in rats (Shaerzadeh et al., 2018). In contrast, brain areas with dopaminergic neuron bodies, such as ventral tegmental area and substantia nigra, show a low sensitivity to the amphetamines-induced microglial activation (Shaerzadeh et al., 2018). Thus, there is compelling evidence indicating decreased neuronal integrity and compromised glial reactivity in the prefrontal cortex (PFC) of human AMPH users and of rats exposed to AMPH (Sailasuta et al., 2010; Mackey et al., 2014; Shaerzadeh et al., 2018). In fact, in rodents and humans, AMPH exposure is usually associated with altered performance in cognitive tasks that are highly dependent on PFC functioning, such as attention and working memory (Potvin et al., 2018; Mizoguchi and Yamada, 2019).

AMPH-induced pro-inflammatory effects extend to brain microvasculature. Indeed, 24 h after non-toxic doses of AMPH, it was observed an increase in pro-inflammatory markers, oxidative stress, and heat shock protein expression in meninges-associated vasculature in rodents (Thomas et al., 2009; Thomas et al., 2010). Likewise, our previous results show a sensitized inflammatory response in brain microvessels 7 days after AMPH withdrawal (Marchese et al., 2020). Moreover, these alterations were accompanied by an angiogenic response in the prelimbic PFC (PL-PFC) and somatosensory cortex, evidenced as an increase in the area occupied by microvessels (Occhieppo et al., 2017; Marchese et al., 2020). The microvessels microarchitecture was also altered, showing fewer branching points and more tortuosity without changes in the diameter.

The renin-angiotensin system (RAS) was initially described as a peripheral humoral system, involved in blood pressure and hydro-electrolyte regulation (Peach 1977). Nowadays, it is recognized as a ubiquitous pleiotropic system, with all of its components synthesized within the central nervous system (von Bohlen and Halbach and Albrecht, 2006). Angiotensin II (Ang II), the main active peptide of the RAS, modulates a wide range of the central nervous system (CNS) functions by its AT_1_-R. Brain AT_1_-R has been reported to play a role in blood pressure and fluid homeostasis regulation, stress responses, depression and cognition, among others (Epstein et al., 1970; Jensen et al., 1992; Raghavendra et al., 2001; Bali and Jaggi 2013). Dopamine-innervated areas express high AT_1_-R density, where they positively modulate dopamine synthesis, tonic, and evoked release (Hoebel et al., 1994; Brown et al., 1996; Paz et al., 2014). In this sense, in previous studies we found that AT_1_-R modulate DA hyperreactivity induced by a single exposure of AMPH in caudate-putamen and nucleus accumbens (Paz et al., 2011). In addition, the previous AT_1_-R blockade prevented the AMPH-induced locomotor sensitization and reversed the psychostimulant-induced locomotor sensitization when these receptors where locally antagonized in the caudate-putamen (Paz et al., 2011; Paz et al., 2014).

Moreover, the presence of AT_1_-R has been described in all the components of the neurovascular unit (Yang et al., 1999; Zhou et al., 2006; Garrido-Gil et al., 2017). AT_1_-R are constitutively expressed in astrocytes and their activation induces increased intracellular Ca^2+^ with the subsequent induction of early gene transcription (Delaney et al., 2008). These receptors regulate astroglial and microglial reactivity, inducing ROS production and pro-inflammatory cytokine release in the presence of lipopolysaccharides (Bhat et al., 2016). In brain microvessels, the activation of AT_1_-R is associated with imbalance in vascular tone regulators together with vascular leakage and inflammatory cell recruitment (Yamakawa et al., 2003; Ando et al., 2004; Zhou et al., 2005).

AT_1_-R blockers are currently used in the antihypertensive treatment and have a low incidence of adverse effects, even in elderly patients and they do not alter blood pressure in normotensive patients (Mimran and Ribstein, 1999; Israili, 2000; Shen et al., 2012). Among the several AT_1_-R blockers available, candesartan (CAND) shows the tightest and longest-lasting binding to AT_1_-R (Unger, 2000). Moreover, it was found that among losartan, irbesartan, telmisartan, CAND was the most effective angiotensin receptor blocker in crossing the blood-brain barrier (Unger, 2003). These reasons and the fact that AT_1_-R activation/stimulation has overlapping effects with AMPH in the neurovascular unit components, make AT_1_-R blockers a potential pharmacological tool to modulate some deleterious effects induced by AMPH. The aim of this study was therefore to evaluate the AT_1_-R role in AMPH-induced oxidative stress and the associated glial and vascular alterations in the PFC. Working and short-term memory were assessed as a functional outcome of PFC performance.

## Materials and Methods

### Animals

Adult male Wistar rats (250–320 g), from the Department of Pharmacology vivarium (Facultad de Ciencias Químicas, Universidad Nacional de Córdoba, Argentina), were randomly housed in groups of three, one week before the beginning of the experimental protocol. The animals were maintained under controlled environmental conditions (20–24°C, 12 h light/dark cycle with lights on at 7 a.m.) with ad libitum access to food and water.

All procedures were approved by the Animal Care and Use Committee of the Facultad de Ciencias Químicas, Universidad Nacional de Córdoba, Argentina (Res. No. 270/18), in accordance with the NIH Guide for the Care and Use of Laboratory Animals.

### Drugs

The selective AT_1_-R antagonist, candesartan cilexetil (CAND, Laboratorios Phoenix, Buenos Aires, Argentina) was dissolved in 0.1 N NaHCO_3_ (vehicle, VEH). The selected dose (3 mg/kg) is in the intermediate doses range (1–5 mg/kg) used in rodents studies (Callera et al., 2016) and was selected from previous work from our laboratory (Paz et al., 2013; Marchese et al., 2016; Occhieppo et al., 2017; Marchese et al., 2020) and taking into account the observed neuroprotective effect at that dose (Lu et al., 2005). d-AMPH sulfate was dissolved in 0.9% NaCl (saline, SAL) immediately before use. The dose (2.5 mg/kg) was selected considering previous studies from our laboratory (Marchese et al., 2016; Marchese et al. 2017; Marchese et al. 2020) and other studies that showed oxidative stress and neuroinflammatory effects induced by AMPH at similar doses (Dean et al., 2011; Gubert et al., 2016; Valvassori et al., 2019).

Animals were administered with the drugs in a different room from that they were housing and from where the behavioral experiments were performed.

### Experimental Design

#### Experimental Protocol 1: AT_1_-R Involvement in AMPH-Induced Oxidative Stress

**FIGURE 1:**
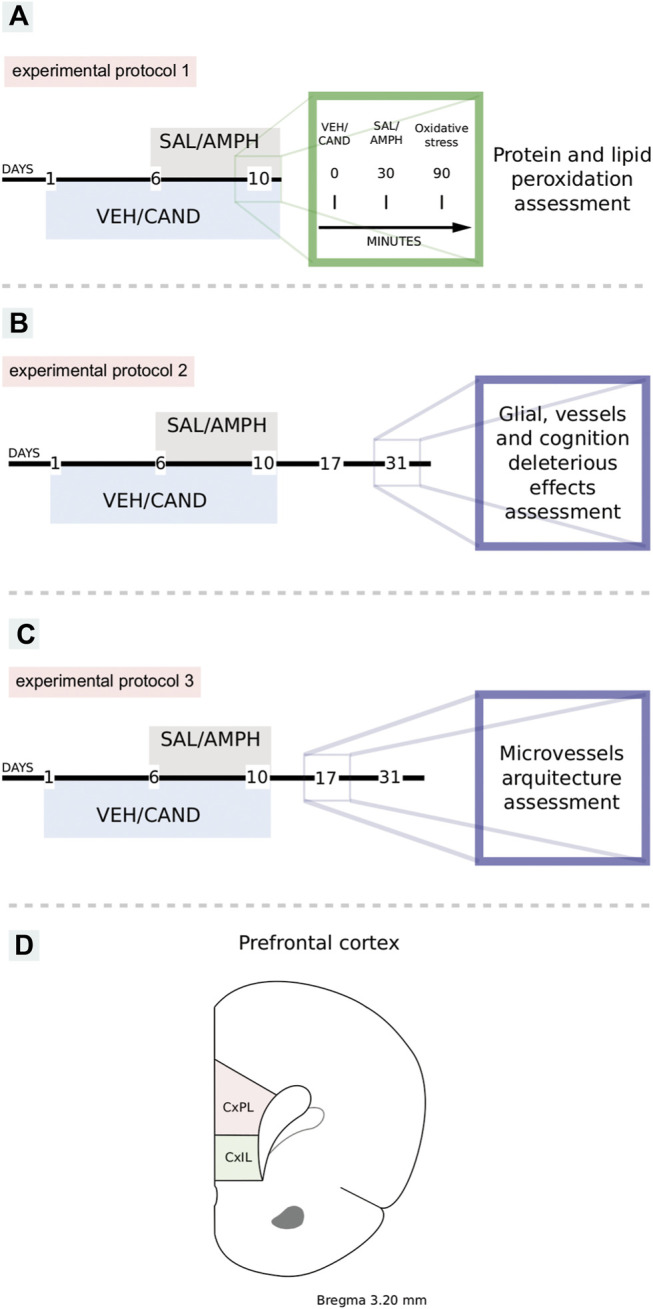
Schematic representation of experimental protocols. VEH: vehicle, CAND: candesartan, SAL: saline, AMPH: amphetamine. Protocol 1: to study the effects of amphetamine and AT1-R blockade in oxidative stress **(A)**. Protocol 2: to study the effects of amphetamine and candesartan on memory, glial reactivity and angioplasticity after 21 days of withdrawal **(B)**. Protocol 3: to study the effects of amphetamine and candesartan on angioplasticity after 7 days of withdrawal **(C)**. Schematic representation of prelimbic and infralimbic subdivisions of the rat prefrontal cortex **(D)**. CxPL: prelimbic prefrontal cortex, CxIL: infralimbic prefrontal cortex.

From days 1 to 5, the animals received an oral dose of VEH/CAND (3 mg/kg) by gavage, using a feeding needle. From day 6 to 10, they received an oral dose of VEH/CAND immediately followed by an injection of SAL/AMPH (2.5 mg/kg, i. p.). Four experimental groups were formed: VEH-SAL, VEH AMPH, CAND-SAL and CAND-AMPH (Figure 1A). On day 10, the drug injection schedule was adapted to measure oxidative stress (see the oxidative stress protocols below).

**GRAPHICAL ABSTRACT:**
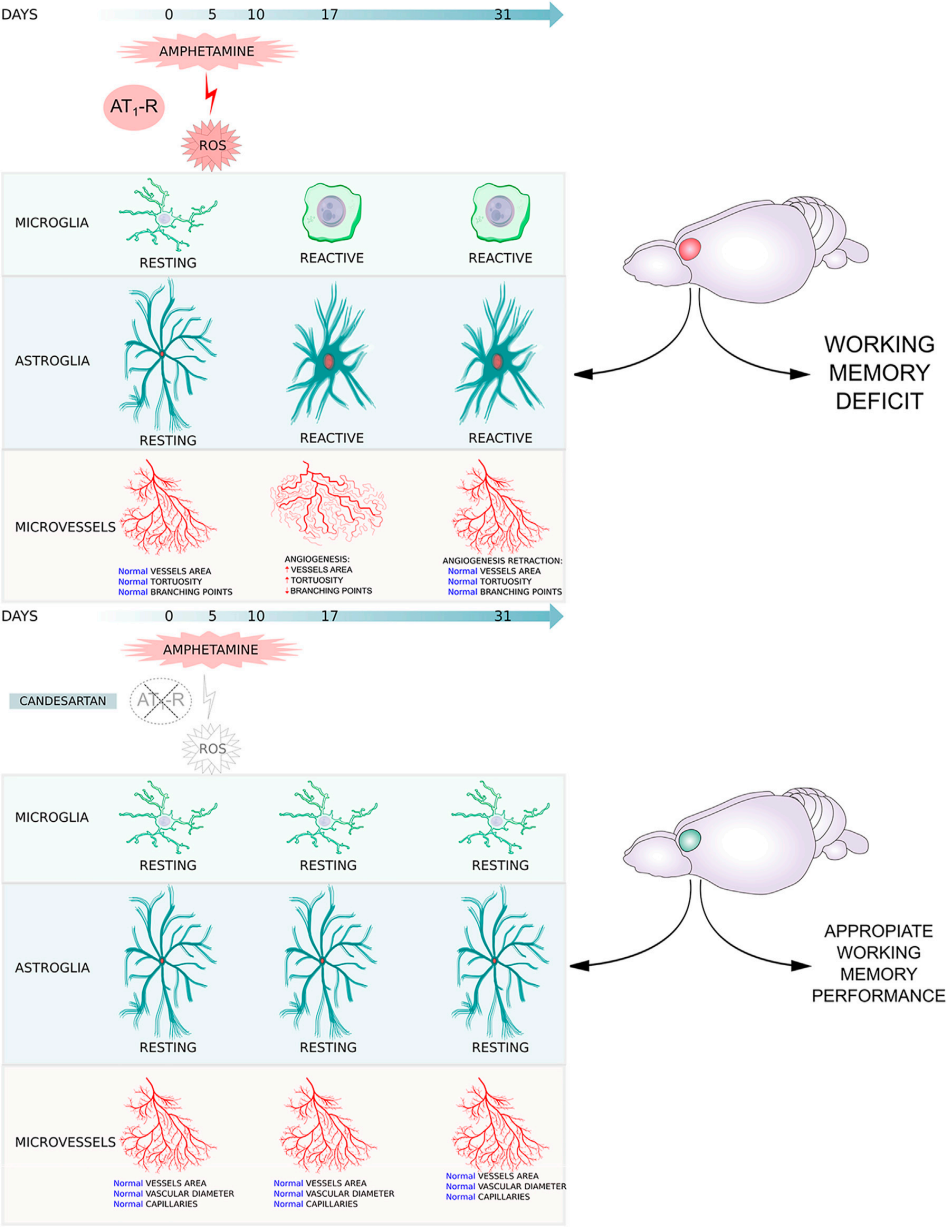


#### Experimental Protocol 2: AT_1_-R Involvement in Amphetamine-Induced Glial Reactivity and Angioplasticity 21 days After Withdrawal

From days 1 to 10, the animals received VEH/CAND (3 mg/kg) by gavage using a feeding needle and from days 6 to 10 after VEH/CAND administration they were injected with SAL/AMPH (2.5 mg/kg, i. p.). They then remained undisturbed in their home cages for a drug-free period of 21 days until the experiment. Four experimental groups were formed: VEH-SAL, VEH-AMPH, CAND-SAL, and CAND-AMPH (Figure 1B).

#### Experimental Protocol 3: AT_1_-R Involvement in Amphetamine-Induced Angioplasticity 7 days After Withdrawal

From days 1 to 10, the animals received VEH/CAND (3 mg/kg) by gavage using a feeding needle and from days 6 to 10 after VEH/CAND administration they were injected with SAL/AMPH (2.5 mg/kg, i. p.). They then remained undisturbed in their home cages for a drug-free period of 7 days until the experiment. Four experimental groups were formed: VEH-SAL, VEH-AMPH, CAND-SAL and CAND-AMPH (Figure 1C).

### Oxidative Stress Assessment by Measurement of Protein and Lipid Peroxidation

On day 10 of experimental protocol 1, the animals received an oral dose of VEH/CAND (3 mg/kg) and 60 min later an injection of SAL/AMPH (2.5 mg/kg), according to their corresponding experimental group. Thirty minutes after the SAL/AMPH injection, the animals were sacrificed by decapitation and PFC was extracted including IL and PL subdivision.

#### Advanced Oxidation Protein Product Quantification

The initial total protein content was detected colorimetrically by Bradford reagent at an absorbance of 595 nm. The subsequent quantification of advanced oxidation protein products (AOPP) was also based on spectrophotometric detection, following Witko-Sarsat et al. (Witko-Sarsat et al., 1996). Briefly, 100 µl of PFC homogenate dilution, 100 µl of chloramine T (ChT, 0–100 µM) for calibration and 200 µl of PBS as blank were applied on a microtiter plate. Then, 10 µl of 1.16 M potassium iodide and 20 µl of glacial acetic acid were added to each well and absorbance at 340 nm was immediately read.

ChT was used as standard for the calibration curve. The concentration of AOPP was expressed as the equivalent of ChT units (µM) per milligram of proteins. All samples were analyzed in duplicate.

#### Lipid Peroxidation

Lipid oxidation was determined following a procedure similar to B. Avci et al. optimized for these samples (Avci et al., 2012). Briefly, 250 µl of trichloroacetic acid and 250 µl of thiobarbituric acid were added to 100 µl of non-diluted homogenate. Immediately, the samples were kept in boiling water for 10 min. Centrifugation at 129 g was performed for 10 min after cooling to clear the supernatant from denaturalized proteins. Absorbance was immediately measured at 532 nm. Thiobarbituric acid reactive substances were quantified using an extinction coefficient of 1.56 × 10^5^ M^−1^ cm^−1^ and expressed as nanomoles of malondialdehyde (MDA) per milligram of proteins. Tissue proteins were estimated using Bradford reagent. All samples were analyzed in duplicate.

### Immunohistochemistry of GFAP, CD11b and von Willebrand Factor

Astrogliosis and microgliosis were assessed by immunolabeling with the mouse monoclonal anti-glial fibrillary acidic protein antibody (GFAP, Sigma-Aldrich, MO, United States) and CD11b (Millipore, CA, United States), respectively. To evaluate the vascular network, endothelial cells were immunolabeled with the rabbit anti-von Willebrand factor (VWF, Dako Denmark A/S).

The animals were anesthetized with urethane (100 mg/kg i.p.) and transcardially perfused with 100 ml of saline and heparin (200 μl/L), followed by 200 ml of 4% paraformaldehyde in 0.1 M PBS (pH 7.4). The brains were removed and stored at 4°C in a 30% sucrose solution. Coronal sections of 20 μm (GFAP and CD11b immunohistochemistry) and 40 μm (VWF immunohistochemistry) were cut using a freezing microtome (Leica CM1510S) and collected in 0.01 M PBS (pH 7.4). Then, the endogenous peroxidase was blocked by incubation in a mixture of 10% H_2_O_2_ and 10% methanol for 2 h, followed by incubation with 10% normal goat serum (NGS; Natocor, Córdoba, Argentina) in 0.1 M PBS for 2 h to block nonspecific binding sites. The free-floating sections were incubated overnight at room temperature with mouse monoclonal GFAP (1:1,000), CD11b (1:1,000) and VWF (1:200). The next day, the sections were rinsed with 0.01 M PBS, and GFAP- and CD11b-labeled sections were incubated with biotin-labeled goat anti-mouse secondary antibody (Jackson Immunoresearch, Laboratories Inc., PA, United States) diluted 1:3,000 in 2% NGS-0.1M PBS and VWF-labeled sections were incubated with biotin-labeled anti-rabbit secondary antibody (Vector Laboratories, CA, United States) diluted 1:500 in 2% NGS-0.1M PBS. Later, they were incubated with avidin-biotin-peroxidase complex diluted 1:500 in 2% NGS-0.1M PBS (ABC-Vector Laboratories, CA, United States) for 2 h each, at room temperature. The peroxidase label was detected with diaminobenzidine hydrochloride (0.5 mg/ml, Sigma-Aldrich, MO, United States) and hydrogen peroxide; the solution was intensified with 1% cobalt chloride and 1% nickel ammonium sulfate. Finally, the free-floating sections were mounted on gelatinized slides, air-dried overnight, dehydrated, cleared in xylene, and placed under a coverslip with DPX mounting medium (Flucka Analytical).

### Image Processing

The images were obtained using a Leica DM4000B microscope equipped with Leica FW4000 and a DFC Leica digital camera attached to a contrast enhancement device, and digitized images were stored in a computer. All the images were obtained with identical exposure times, gain and offsets, and saved in TIFF format (1392 × 1040 pixels). The images were processed using ImageJ software (Schindelin et al., 2012). The analyses were made blinded to the experimental groups.

Image quantification was performed in the infralimbic (IL-PFC) and prelimbic (PL-PFC) areas (Bregma: 3.20 mm), which were identified and delimited according to the Paxinos and Watson atlas 2009 (both subregions are schematized in Figure 1D). The measurements were taken bilaterally in two sections and the final value was obtained as the average of the four sections counted.

#### Astrocyte Reactivity by GFAP Immunostaining

GFAP-stained sections were taken at 400x magnification and the area occupied by astrocytes was quantified fixing a threshold of 140–180 and expressed as the proportion of total area, evaluated in percentages.

#### Microglia Reactivity by CD11b Immunostaining

CD11b-stained sections were taken at 400x magnification and the area occupied by microglia was quantified fixing a threshold of 130–150 and expressed as the proportion of total area, evaluated in percentages.

#### Skeleton Analysis of Glial Cells

Glial morphology was assessed following a procedure adapted from Morrison et al. (Morrison and Filosa, 2013). Photomicrographs were pre-processed by the following workflow process: background subtraction, selection of the regions of interest with the multi-point tool, appling the morphological reconstruction tool of the MorpholibJ plugin (Legland et al., 2016), binarize the image by threshold selection and skeletonize the binary images. The AnalizeSkeleton plugin of ImageJ software (Arganda-Carreras et al., 2010) was applied and the total number of branches and branches average length was calculated. The number of branches was normalized dividing by the total number of pixels.

#### Vascular Network by von Willebrand Factor Immunostaining

The cortical microvessel architecture was analyzed by VWF immunohistochemistry using the vessel analysis plugin of ImageJ software (Elfarnawany, 2015), adapted from previous reports (Tata and Anderson 2002; Beauquis et al., 2010; Marchese et al., 2017; Marchese et al., 2020; Occhieppo et al., 2020). The parameters assessed were:

ߦ Percentage of vessel area.
Number of branching points in 0.01 mm^2^ of VWF positive area.
ߦ Vessel tortuosity (ratio between the real distance of adjacent branching points and their Euclidean distance—the shortest distance between them). The latter parameter takes values from 1 to infinite, where higher values indicate a more sinuous structure.

### Behavior

#### Y-Maze Test

**FIGURE 2:**
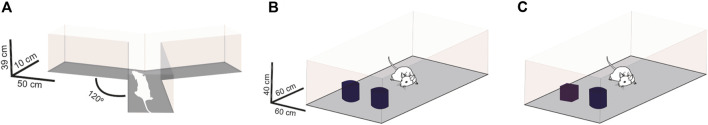
Schematic representation of working memory and short-term memory assessment. Working memory assessment by Y-maze test **(A)**. Sample session of novel object recognition test **(B)**. Test session of novel object recognition test **(C)**.

Working memory was assessed by the Y-maze test adapted from previous reports (Ahmad-Molaei et al., 2018; Famitafreshi and Karimian, 2018; Islam et al., 2019; Marchese et al., 2020). Briefly, the animals were placed at the center of a Y-shaped maze, with three equal arms (50 × 10 cm × 39 cm, at angles of 120°) and left to explore freely for 8 min (Figure 2A). The trial was monitored through a video camera positioned above the Y-maze and the number of spontaneous alternations and arms entries was counted. An arm entry was defined when four paws were within the arm. Spontaneous alternation was defined as three consecutive choices of three different arms. The results were expressed as the ratio between spontaneous alternation and the total possible alternations (number of entries minus 2). In this behavioral task, a lower alternation percentage is considered as a working memory deficit. Animals that displayed a 2 min period of immobility between arms were excluded from the final analysis.

#### Novel Object Recognition Test

Short-term memory was evaluated by the novel recognition test adapted from previous works (Baker and Kim, 2002; Arias-Cavieres et al., 2017; Ahmad-Molaei et al., 2018). Briefly, the animals were placed in an empty open arena (60 × 60 × 40 cm) in a different room from where they were housed. All the rats were tested between 9 a.m. and 6 p.m., under dim light, in a quiet room. The day before the experiment, the animals were habituated individually in the testing apparatus for 5 min, in two sessions 2 h apart. At the sample session on the experimental day, the animals were placed in the open arena, which had two identical objects aligned with a random wall of the arena, at a distance of 10 cm, and they were allowed to freely explore for 5 min (Figure 2B). In the test session 2 h later, the animals were placed in the same arena, but one object of the sample session had been replaced by a new one that had a different shape and texture (Figure 2C). Trials were monitored through a video camera positioned above each arena and the time spent in contact with the objects was quantified in both sessions. The results were expressed as the preference index (PI) of the new object (test session) and PI of the most explored object (sample session), calculated as follows:

$$PI=\frac{\text{novelobjectexploration}}{\text{totalobjectexploration}}\left(\frac{s}{s}\right).$$

A PI greater than 0.5 in the test session shows a preference for the new object and therefore recognition of the sample session objects. A PI near to 0.5 in the sample session shows no bias in the training session.

Two identical sets of different objects were used made of glass and plastic with variations in shape and texture.

### Statistical Analysis

The data were analyzed using two-way ANOVA and reported as means ± SEM. The analysis considered VEH/CAND as a treatment factor and SAL/AMPH as a drug factor. If interaction was observed, multiple comparisons were made using the Tukey post-test. A value of *p* < 0.05 was considered significant. The analyses were performed using Graphpad Prism® 8.03 software.

## Results

### AT_1_-R Blockade Prevents Amphetamine-Induced Oxidative Stress

**FIGURE 3:**
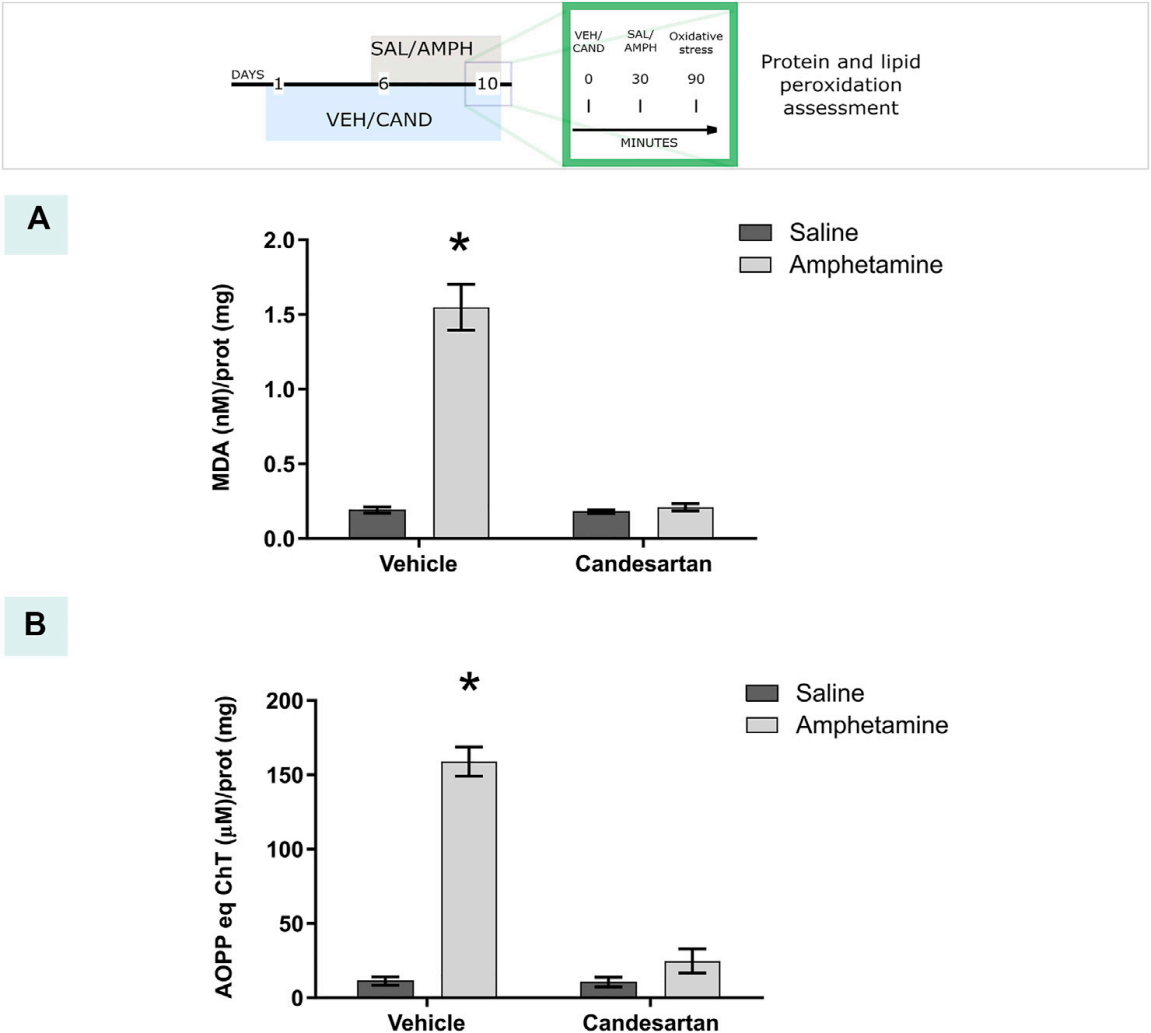
AT_1_-R blockade prevents amphetamine-induced oxidative stress. The graphs show the MDA and AOPP quantification as measurements of lipid and protein peroxidation, respectively, in the PFC for the four experimental groups of protocol 1. MDA quantification **(A)**. Equivalents of chloramine T quantification **(B)**. Values are the mean ± SEM; *n* = 4. *different from the rest of the groups (*p* < 0.05).

Acute AMPH exposure induced an extensive oxidative stress, evaluated as lipid and protein peroxidation, which was totally blunted by the AT_1_-R blockade pretreatment (Figure 3, experimental protocol 1).

The two-way ANOVA results of protein peroxidation showed a significant effect of treatment, drug and drug * treatment interaction factors (F_(1, 12)_ = 145.9, *p* < 0.01; F_(1, 12)_ = 101.4, *p* < 0.01; F_(1, 12)_ = 99.29, *p* < 0.01 respectively). Tukey Post Hoc comparison indicates a significant increase of protein peroxidation in the PFC in VEH-AMPH group when was compared with the rest of the groups (*p* < 0.01) and no significant differences in the comparison between the control groups (VEH-SAL and CAND-SAL; *p* > 0.99) and control groups with CAND-AMPH (*p* = 0.51 and *p* = 0.47, respectively, Figure 3A).

The two-way ANOVA results of lipid peroxidation showed a significant effect of treatment, drug and drug * treatment interaction factors (F_(1, 12)_ = 78.15, *p* < 0.01; F_(1, 12)_ = 74.09, *p* < 0.01; F_(1, 12)_ = 71.75, *p* < 0.01 respectively). Tukey Post Hoc comparison indicates a significant increase of lipid peroxidation in the PFC in VEH-AMPH group when was compared with the rest of the groups (*p* < 0.01) and no significant differences in the comparison between the control groups (VEH-SAL and CAND-SAL; *p* > 0.99) and control groups with CAND-AMPH (*p* > 0.99 and *p* > 0.99, respectively, Figure 3B).

### Amphetamine-Induced Gliosis in the Prelimbic Prefrontal Cortex Involves AT_1_-R

**FIGURE 4:**
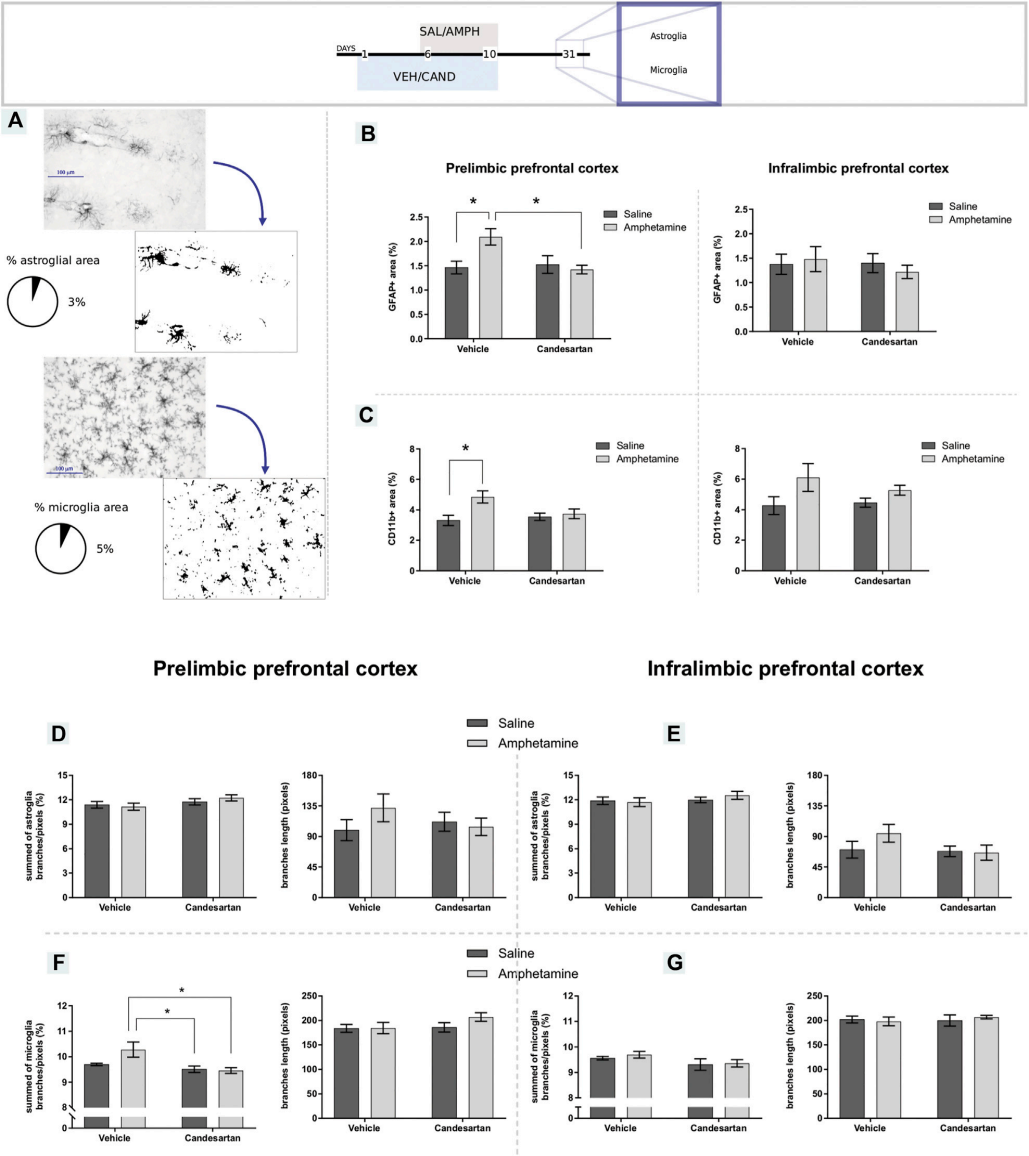
Amphetamine induces gliosis selectively in the prelimbic prefrontal cortex by AT_1_-R. The graphs show GFAP and CD11b expression and glial morphology in the PL- and IL-PFC as a measure of astrogliosis and microgliosis, respectively, for the four experimental groups of protocol 3. Schematic representation of GFAP and CD11b immunostaining assessment **(A)**. GFAP immunoreactive area in the PL- and IL-PFC **(B)**, left and right panel, respectively. CD11b positive area in the PL- and IL-PFC **(C)**, left and right panel, respectively. Summed of astroglia branches and average branches length in PL-PFC **(D)**, left and right panel, respectively. Summed of astroglia branches and average branches length in IL-PFC **(E)**, left and right panel, respectively. Summed of microglia branches and average branches length in PL-PFC **(F)**, left and right panel, respectively. Summed of microglia branches and average branches length in IL-.PFC **(G)**, left and right panel, respectively. Values are the mean ± SEM; *n* = 6–9. *different from VEH-SAL and CAND-AMPH (*p* < 0.05; Figure 4A) or different from VEH-SAL (*p* < 0.05; Figure 4B) or CAND-SAL and CAND-AMPH (*p* < 0.05; Figure 4F panel left).

Glial reactivity (astrogliosis and microgliosis) was observed after AMPH exposure in the PL-PFC, but not in the IL-PFC. These alterations were prevented by AT_1_-R blockade pretreatment (Figures 4B,C). In PL-PFC, AMPH exposure increased the ramification of microglia and this alteration was prevented by the AT_1_-R blockade. No changes in microglia ramification were observed in IL-PFC or in astroglia ramification in PL and IL-PFC after the treatments. The average branches length was similar among groups in microglia and astroglia in both brain regions.

The two-way ANOVA results of GFAP immunostaining in PL-PFC showed a significant effect of treatment and drug * treatment interaction factors (F_(1, 28)_ = 4.29, *p* = 0.048; F_(1, 28)_ = 6.12, *p* = 0.020; respectively) and no significant effect of drug factor (F_(1, 28)_ = 3.15, *p* = 0.087). Tukey Post Hoc comparison indicates a significant increase of GFAP occupied area in the PL-PFC in VEH-AMPH group when was compared with the VEH-SAL and CAND-AMPH groups (*p* = 0.041 and *p* = 0.022, respectively) and no significant differences in the comparison between the control groups (VEH-SAL and CAND-SAL; *p* > 0.99) and control groups with CAND-AMPH (*p* > 0.99 and *p* = 0.95, respectively; Figure 4B, left panel).

The two-way ANOVA results of CD11b immunostaining in PL-PFC showed a significant effect of drug and drug * treatment interaction factors (F_(1, 28)_ = 7.25, *p* = 0.012; F_(1, 28)_ = 4.38, *p* = 0.046; respectively) and no significant effect of treatment factor (F_(1, 28)_ = 1.83, *p* = 0.187). Tukey Post Hoc comparison indicates a significant increase of CD11b occupied area in the PL-PFC in VEH-AMPH group compared with the VEH-SAL and CAND-SAL groups (*p* = 0.015 and *p* = 0.048, respectively) and no significant differences in the comparison between control groups (VEH-SAL and CAND-SAL; *p* = 0.94) and VEH-AMPH and CAND-AMPH groups (*p* = 0.126; Figure 4C, left panel).

The two-way ANOVA results of GFAP immunostaining in IL-PFC showed no significant effect of drug, treatment and drug * treatment interaction factors (F _(1, 25)_ = 0.03, p = 0.849: F _(1, 25)_ = 0.34, p = 0.563; F _(1, 25)_ = 0.50, p = 0.486; respectively; Figure 4B right panel). The two-way ANOVA results of CD11b immunostaining in IL-PFC showed a significant effect of drug (F _(1, 21)_ = 6.57, p = 0.018) and no significant effects of treatment and drug*treatment interaction factors (F _(1, 21)_ = 0.38, p = 0.542: F _(1, 21)_ = 0.97, p = 0.335; respectively; Figure 4C right panel).

The two-way ANOVA results of microglia ramification in PL-PFC showed a significant effect of treatment and drug * treatment interaction factors (F_(1, 28)_ = 11.70, *p* = 0.002; F_(1, 28)_ = 4.63, *p* = 0.040; respectively) and no significant effect of drug factor (F_(1, 28)_ = 3.19, *p* = 0.085). Tukey Post Hoc comparison indicates a significant increase of microglia ramification in the PL-PFC in VEH-AMPH group compared with the CAND-SAL and CAND-AMPH groups (*p* = 0.007 and *p* = 0.005, respectively) and no significant differences in the comparison between control groups (VEH-SAL and CAND-SAL; *p* = 0.771) and VEH-AMPH and VEH-SAL groups (*p* = 0.056; Figure 4F, left panel).

The two-way ANOVA of microglia ramification in IL-PFC and astroglia ramification in both brain areas and average branches length of microglia and astroglia in both areas showed no significant effects in drug, treatment and drug * treatment interaction factors. These results are summarized in Table 1 and in Figures 4D–G.

**TABLE 1:** Statistical results of glial morphological changes in PL and IL-PFC.

Prelimbic prefrontal cortex								
2-Way ANOVA	Microglia branches lenght (pixels)		Summed of astroglia branches/pixels (%)		Astroglia branches lenght (pixels)	
drug * treatment interaction	F_(1, 28)_ = 1.154	*p* = 0.292	F_(1, 27)_ = 0.764	*p* = 0.390	F_(1, 27)_ = 1.692	*p* = 0.204
Treatment	F_(1, 28)_ = 1.702	*P* = 0.203	F_(1, 27)_ = 3.161	*p* = 0.087	F_(1, 27)_ = 0.257	*P* = 0.616
Drug	F_(1, 28)_ = 1.317	*p* = 0.261	F_(1, 27)_ = 0.089	*p* = 0.768	F_(1, 27)_ = 0.678	*p* = 0.418	

Infralimbic prefrontal cortex								
2-Way ANOVA	Summed of microglia branches/pixels (%)		Microglia branches lenght (*p*ixels)		Summed of astroglia branches/*p*ixels (%)		Astroglia branches lenght (*p*ixels)	
drug * treatment interaction	F_(1, 19)_ = 0.067	*p* = 0.799	F_(1, 21)_ = 0.398	*p* = 0.535	F_(1, 27)_ = 0.614	*p* = 0.440	F_(1, 27)_ = 1.343	*p* = 0.257
treatment	F_(1, 19)_ = 3.337	*p* = 0.084	F_(1, 21)_ = 0.154	*p* = 0.699	F_(1, 27)_ = 1.027	*p* = 0.320	F_(1, 27)_ = 1.893	*p* = 0.180
drug	F_(1, 19)_ = 0.326	*p* = 0.575	F_(1, 21)_ = 0.029	*p* = 0.865	F_(1, 27)_ = 0.178	*p* = 0.676	F_(1, 27)_ = 0.957	*p* = 0.337

### Amphetamine Induces Transient Angiogenesis in the Prefrontal Cortex

**FIGURE 5:**
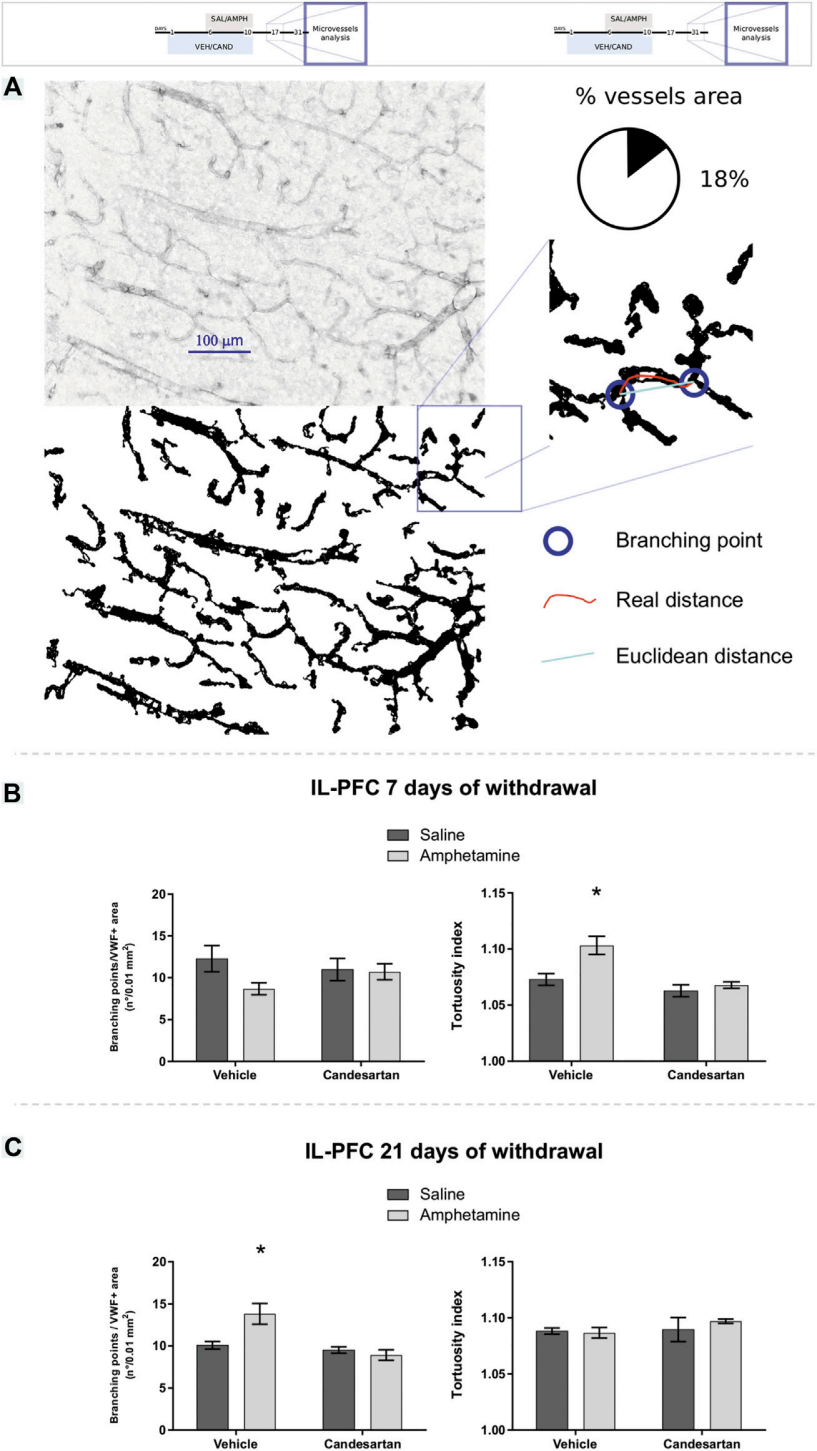
Amphetamine induces transient angiogenesis in the prefrontal cortex. The graphs show VWF expression in the PL-PFC and IL-PFC as a measure of angioplasticity for the four experimental groups of experimental protocols 2 and 3. Schematic representation of the VWF immunostaining assessment **(A)**. The number of branching points and vascular tortuosity in IL-PFC after 7 days of withdrawal (experimental protocol 2) **(B)**, left and right panel, respectively. The number of branching points and vascular tortuosity in IL-PFC after 21 days of withdrawal (experimental protocol 3) **(C)**, left and right panel, respectively. Values are the mean ± SEM; *n* = 5–8. *different from VEH-SAL and CAND-AMPH (*p* < 0.05).

In IL-PFC, after 7 days of withdrawal, it was found an increase of microvessels tortuosity induced by AMPH exposure, that was prevented by AT_1_-R blockade and no changes were observed in branching points microvessels (Figure 5B). On the contrary, after 21 days of withdrawal, it was observed no changes in microvessels tortuosity and an increase of branching points induced by AMPH in this brain area (this alteration was prevented by AT_1_-R blockade, Figure 5C). No changes were observed in all microvessels parameters analyzed in PL-PFC after 21 days of withdrawal (Table 2).

The two-way ANOVA results of microvessels tortuosity in IL-PFC after 7 days of withdrawal showed a significant effect of drug, treatment and drug*treatment interaction factors (F _(1, 27)_ = 9.22, *p* = 0.005; F _(1, 27)_ = 15.17, *p* < 0.001; F _(1, 27)_ = 4.67, *p* = 0.040; respectively; Figure 5B right panel). Tukey Post Hoc comparison indicates a significant increase of microvessels tortuosity in the IL-PFC of VEH-AMPH group compared with the VEH-SAL, CAND-SAL and CAND-AMPH groups (*p* = 0.005, *p* < 0.001, and *p* = 0.001, respectively) and no significant differences in the comparison between control groups (VEH-SAL and CAND-SAL; *p* = 0.604) and CAND-SAL and CAND-AMPH groups (*p* = 0.929; Figure 5B, right panel). The two-way ANOVA results of microvessels branching points in IL-PFC after 7 days of withdrawal showed no significant effect of drug, treatment and drug * treatment interaction factors (F _(1, 27)_ = 2.59, *p* = 0.119; F _(1, 27)_ = 0.09, *p* = 0.760; F _(1, 27)_ = 1.92, *p* = 0.178; respectively; Figure 5B left panel).

The two-way ANOVA results of tortuosity in IL-PFC after 21 days of withdrawal showed no significant effects in drug (F_(1, 18)_ = 0.27, *p* = 0.601), treatment (F _(1, 18)_ = 1.08, *p* = 0.313) and drug * treatment interaction factors (F _(1, 18)_ = 0.64, *p* = 0.432; Figure 5C right panel). The two-way ANOVA results of branching points microvessels in IL-PFC after 21 days of withdrawal showed a significant effects of drug, treatment of drug * treatment interaction factors (F _(1, 18)_ = 4.77, *p* = 0.042; F _(1, 18)_ = 14.54, *p* = 0.001; F _(1, 18)_ = 9.08, *p* = 0.007; respectively; Figure 5C left panel). Tukey Post Hoc comparison indicates a significant increase of branching points in the IL-PFC of VEH-AMPH group compared with the VEH-SAL, CAND-SAL and CAND-AMPH groups (*p* = 0.008, *p* = 0.004 and *p* < 0.001, respectively) and no significant differences in the comparison between control groups (VEH-SAL and CAND-SAL; *p* = 0.942) and CAND-SAL and CAND-AMPH groups (*p* = 0.935; Figure 5C).

The two-way ANOVA results of microvessels occupied area, branching points and tortuosity in PL-PFC after 21 days of withdrawal showed no significant effect of drug, treatment and drug * treatment interaction factors (Table 2).

**TABLE 2:** Statistical results of vascular morphological changes in PL-PFC.

Prelimbic prefrontal cortex						
2-Way ANOVA	Branching points/area		Tortuosity index		vWF occupied area %	
VEH - SAL	5.76 ± 0.64		1.068 ± 0.004		7.27 ± 1.12	
VEH - AMPH	7.39 ± 1.60		1.077 ± 0.006		10.57 ± 2.08	
CAND - SAL	5.64 ± 1.46		1.080 ± 0.003		7.91 ± 0.98	
CAND - AMPH	4.26 ± 0.80		1.074 ± 0.002		7.84 ± 0.64	
drug*treatment interaction	F _(1, 19)_ = 1.681	p = 0.210	F _(1, 19)_ = 3.455	p = 0.079	F _(1, 19)_ = 1.862	P = 0.188
Treatment	F _(1, 19)_ = 1.956	p = 0.178	F _(1, 19)_ = 1.272	p = 0.273	F _(1, 19)_ = 0.722	P = 0.406
Drug	F_(1, 19)_ = 0.012	p = 0.913	F _(1, 19)_ = 0.043	p = 0.838	F _(1, 19)_ = 1.714	P = 0.206

### Amphetamine-Induced Spatial Working Memory Deficit Involves AT_1_-R

**FIGURE 6:**
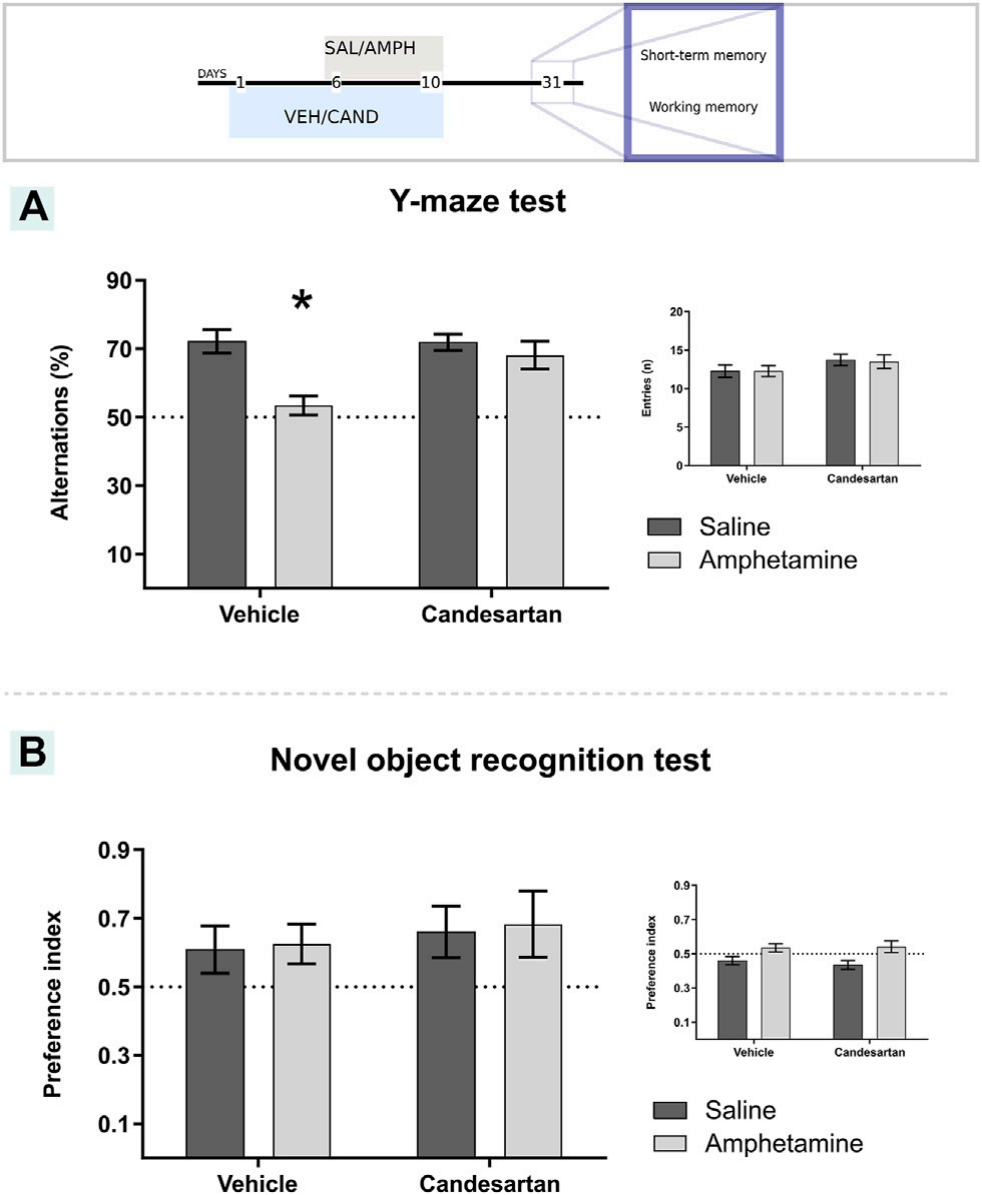
Candesartan prevented amphetamine-induced working memory deficit and amphetamine did not induce changes in short-memory recognition performance. The graph shows working memory performance assessed by the Y-maze paradigm **(A)**. The main graph shows the percentage of spontaneous alternations in a Y-maze arena for the four experimental groups of protocol 3. Inset graph shows the number of entries to each arm in the Y-maze test. Values are the mean ± SEM, *different from the rest of the groups (p < 0.05); n = 10-11. The graph shows short-term memory performance evaluated as the time exploring each object in the one trial recognition task **(B)**. The results were expressed as the preference index for the four experimental groups in the test session of protocol 3. The inset figure shows the preference index for the sample session. Values are the mean ± SEM; n = 7-8.

Previous exposure to AMPH induced a spatial working memory deficit, assessed after 21 days of withdrawal in the Y-maze paradigm (Figure 6A; experimental protocol 3). This deficit was not observed with the CAND pretreatment (Figure 6A; experimental protocol 3).

The two-way ANOVA results of spatial working memory assessment (Y-maze test) showed a significant effect of drug, treatment and drug * treatment interaction factors (F_(1, 39)_ = 12.51, *p* = 0.001; F_(1, 39)_ = 5.11, *p* = 0.029; F_(1, 39)_ = 5.50, *p* = 0.024; respectively; Figure 6A). Tukey Post Hoc comparison indicates a significant lower percentage of spontaneous alternations of VEH-AMPH group compared with the VEH-SAL, CAND-SAL and CAND-AMPH groups (*p* < 0.001, *p* = 0.001 and *p* = 0.013, respectively) and no significant differences in the comparison between control groups (VEH-SAL and CAND-SAL; *p* > 0.999) and CAND-SAL and CAND-AMPH groups (*p* = 0.838; Figure 6A).

An additional analysis of the total activity during the behavioral test was performed to discard motor differences among the four experimental groups (inset graph in Figure 6A). There were no differences in total number of entries to the Y-maze arms among drug, treatment and drug*treatment interaction factors (F_(1, 39)_ = 0.02, *p* = 0.886, F_(1, 39)_ = 2.90, *p* = 0.096, F_(1, 39)_ = 0.02, *p* = 0.886; respectively; inset Figure 6A).

### Amphetamine Does Not Alter Short-Term Recognition Memory

AMPH exposure did not alter short-term recognition memory assessed in the novel object recognition (NOR) paradigm (Figure 6B; Experimental protocol 3). In the test session, the animals from the four experimental groups showed a greater preference for the new object, with a PI for the novel object greater than 0.5 (which shows recognition of the objects of the sample session) with no differences among the groups in drug, treatment and treatment*drug interaction factors (F_(1, 26)_ = 0.07, *p* = 0.796; F_(1, 26)_ = 0.53 *p* = 0.474; F_(1, 26)_ < 0.01, *p* = 0.965; respectively). Additionally, the PI of the sample test was around 0.5 for the four experimental groups with no differences in treatment and drug*treatment interaction factors (F_(1, 26)_ = 0.13 *p* = 0.725; F_(1, 26)_ = 0.35, *p* = 0.558; respectively; inset graph in Figure 6B). A significant effect of drug factor was observed (F_(1, 26)_ = 11.30, *p* = 0.002).

## Discussion

This work shows that AT_1_-R blockade prevents AMPH-induced oxidative stress, evaluated as lipid and protein peroxidation. Our findings also support an AT_1_-R role in the AMPH-induced transient angiogenesis in PFC and long-lasting astroglial and microglial reactivity (evaluated after 21 off-drug days) in the PL-PFC. Furthermore, we showed that AMPH, via AT_1_-R, induced an enduring working memory deficit without short-term memory deleterious effects.

Several sources of brain insults induce oxidative stress via AT_1_-R. Goel et al. and Salmani et al. showed that brain oxidative stress induced by central or peripheral LPS injection is modulated by AT_1_-R, preventing its development by previous CAND or losartan oral administration (Goel et al., 2018; Salmani et al., 2020). Moreover, it has been shown that CAND at low doses (0.1 and 0.3 mg/kg) attenuate ischemic brain damage and inhibit the associated oxidative stress (Hamai et al., 2006). Moreover, Bild et al. showed that central angiotensin II injection (*via* i.c.v.) induced oxidative stress, which is prevented by central injection of AT_1_-R blockers or angiotensin-converting enzyme inhibitors (Bild et al., 2013). In line with the present results, Xu et al. showed that methamphetamine induces oxidative stress and the associated neurotoxicity by AT_1_-R, via phospholipase C β1 (Xu et al., 2021). As oxidative stress plays a crucial role in the amphetamines-induced neurotoxicity (Moratalla et al., 2017), the above and the present results suggest that AT_1_-R may be involved in neurotoxicity effects of others amphetamines derivatives such as 3,4-methylenedioxymethamphetamine. The above described may result from AT_1_-R regulation of mitochondria functioning (the main source of ROS). Recently, Valenzuela et al. showed that AT_1_-R are present in this organelle and that their activation increases superoxide production by inducing NADPH oxidase complex activity (Valenzuela et al., 2016). Ang II also enhances cytoplasmic Ca^2+^, which further activates NADPH oxidase complex activity (Prusty et al., 2017). The resulting oxidative damage may trigger the glial activation in PL-PFC observed 21 days after the last AMPH exposure. Furthermore, the prevention of glial reactivity via AT_1_-R may be a direct effect, since these receptors are present in both cell types (Garrido-Gil et al., 2017). The increased CD11b positive area induced by AMPH exposure, through AT_1_-R, in PL-PFC could result from the observed increase in microglia ramification. Morrison and Filosa reported a similar phenomenom, where ischemic stroke induced an increase of CD11b expression only in brain areas where an hyper-ramification microglia was presented (Morrison and Filosa, 2013). On the contrary, since no morphological changes were observed in astroglia, the increased GFAP positive area could be related with others processes such as increased number of GFAP positive cells or an increased tortuosity in their ramifications.

It was shown that AT_1_-R blockade exerts protective effects over gliosis and the release of pro-inflammatory compounds in several animal models of neuroinflammation (Pang et al., 2012; Labandeira-Garcia et al., 2017; Trigiani et al., 2018; Gong et al., 2019; Subudhi et al., 2019). Besides the inhibition of AT_1_-R signaling, the anti-inflammatory effects of AT_1_-R blockers could be also related to the shifting of Ang II effects towards AT_2_ receptors (Gebre et al., 2018). In this regard, Bhat et al. showed that LPS increase AT_1_-R and decrease AT_2_-R expression in both *in vitro* and *in vivo* studies and the CAND prevention of the LPS-induced inflammation in both models is abolished when CAND is co-administered with an AT_2_-R blocker (PD_123319_) (Bhat et al., 2016). Even though CAND is accepted as a weak partial gamma receptor agonist (in contrast with others AT_1_-R antagonists such as telmisartan and irbesartan) (Michel et al., 2013), the described modulation of glial reactivity by CAND could be related to the modulation of these receptors, as it was described in an animal model of traumatic brain injury (Villapol et al., 2012).

Despite their substantial functional differences, the IL- and the PL-PFC are often considered as a single brain region (ventral medial PFC) (Vertes, 2004). While IL-PFC functioning is involved with visceral/autonomic activity, the PL-PFC is linked with cognitive processes such as attention and working memory (Vertes, 2004). The PL-PFC seems to be particularly sensitive to AMPH damage since no glial changes were observed in the IL-PFC. These results are in line with our previous work showing AT_1_-R participation in PFC microglial and astroglial reactivity 7 days after AMPH withdrawal. In agreement with our previous studies, these results showed no AMPH-induced alteration in IL-PFC glial reactivity (Marchese et al., 2020). Bull et al. reported a similar astrocyte response, showing increased GFAP expression in the PL-PFC but not in the IL-PFC subdivision, after alcohol withdrawal (Bull et al., 2015). The differences between these brain regions may thus extend to AMPH toxicity. The selectiveness of the AMPH damage for discrete brain regions may explain the alteration of working memory but not of short-term memory observed in this work. It is well known that working memory depend on an integrated activity between PL-PFC and hippocampus (Spellman et al., 2015), while short-term memory mainly depends on the hippocampus and sub-regions of the medial temporal lobe such as the entorhinal and perirhinal cortex (Cohen and Stackman, 2015). In previous work, we observed an altered working memory performance together with astrogliosis and microgliosis in PL-PFC without glial alterations in dentate gyrus of hippocampus after 7 days of AMPH withdrawal, using the same psychostimulant exposure protocol (Marchese et al., 2020). Although, in the present work the glial reactivity was not evaluated after 21 days of withdrawal in hippocampus, the above results suggest that AMPH induces working memory independently of glial hippocampus alterations and co-occur with PL-PFC gliosis. The AMPH-induced disturbance in the PL-PFC that leads to microglial and astroglial activation may also induce dysfunction in this brain area, leading to working memory deficit. Moreover, sustained glial activation may induce a neuroinflammatory scenario and contribute to PL-PFC dysfunction, in a self-perpetuating loop of damage-glial activation-neuroinflammation. In this regard, de Souza Gomez et al. reported that CAND prevents the working memory deficit and the oxidative imbalance induced by AMPH in mice assessed after a short withdrawal period (2 h) in the Y-maze paradigm (de Souza Gomes et al., 2015). In line with the present results, Che et al. and Arroyo-Garcia et al. reported no significant effects of AMPH exposure in short-term memory performance assessed in novel object recognition after prolonged withdrawal (more than 10 days) (Che et al., 2013; Arroyo-García et al., 2020). Despite that, all experimental groups showed a greater preference for the new object in the test session, the significant lower PI of control groups in the sample session could be a bias factor, affecting the test session performance in these experimental groups.

In previous studies, we showed that after 7 days withdrawal, AMPH induces angiogenesis in the PL-PFC via AT_1_-R (Marchese et al., 2020). Interestingly, in the present work, we found no changes in PL-PFC microvessels area 21 days after AMPH withdrawal, indicating that vessel pruning took place after day 7 of withdrawal. Furthermore, other microvessel features such as branching points and tortuosity were also normalized 21 days after withdrawal. Considering that microvascular architecture were normalized after 21 days of withdrawal and working memory remained altered; the angiogenic changes observed in previous work after 7 days of AMPH withdrawal in PL-PFC (Marchese et al., 2020) would appear as a compensatory mechanism of hypoxia and/or inflammatory state induced by the psychostimulant and would not contribute to AMPH-induced working memory deficit. Moreover, the above results suggest that AMPH induces an angiogenic scenario via AT_1_-R that gradually returns to normal in absence of the psychostimulant. Similar results were observed when rodents were exposed to prolonged hypoxia, showing increased angiogenesis that was later normalized in a normoxic environment (LaManna 2012). It has been shown that AT_1_-R are involved in neovascularization in differents animals models of angiogenesis. In this sense, Miyajima et al. reported that the AT_1_-R blockade by CAND prevents the neovascularization in a mouse renal cancer lung metastasis, preventing the increase in the CD34 and VEGF expresion in the tumor (Miyajima et al., 2002). Moreover, Kurosaka et al. showed a reduced angiogenesis (reduced CD31-positive microvessels) in AT_1_-R knockout mice and in mice treated with the AT_1_-R antagonist TCV-116 in a mouse model of wound healing (Kurosaka et al., 2009). Proangiogenic role of AT_1_-R was also reported in an angiogenesis Matrigel model in mice, where the AT1-R blockade by CAND prevents the angiogenesis induction by the Matrigel injection measured by the CD31 positive cells count (Tamarat et al., 2002). Furthermore, Chu et al., using a similar AMPH protocol (2 mg/kg i.p. for four days), reported an increase of hypothalamic HIF-1, the main mediator of angiogenesis (Chu et al., 2019). Therefore, AMPH may induce angiogenic signals, even under normoxic conditions, leading to the transient angiogenesis observed.

**FIGURE 7:**
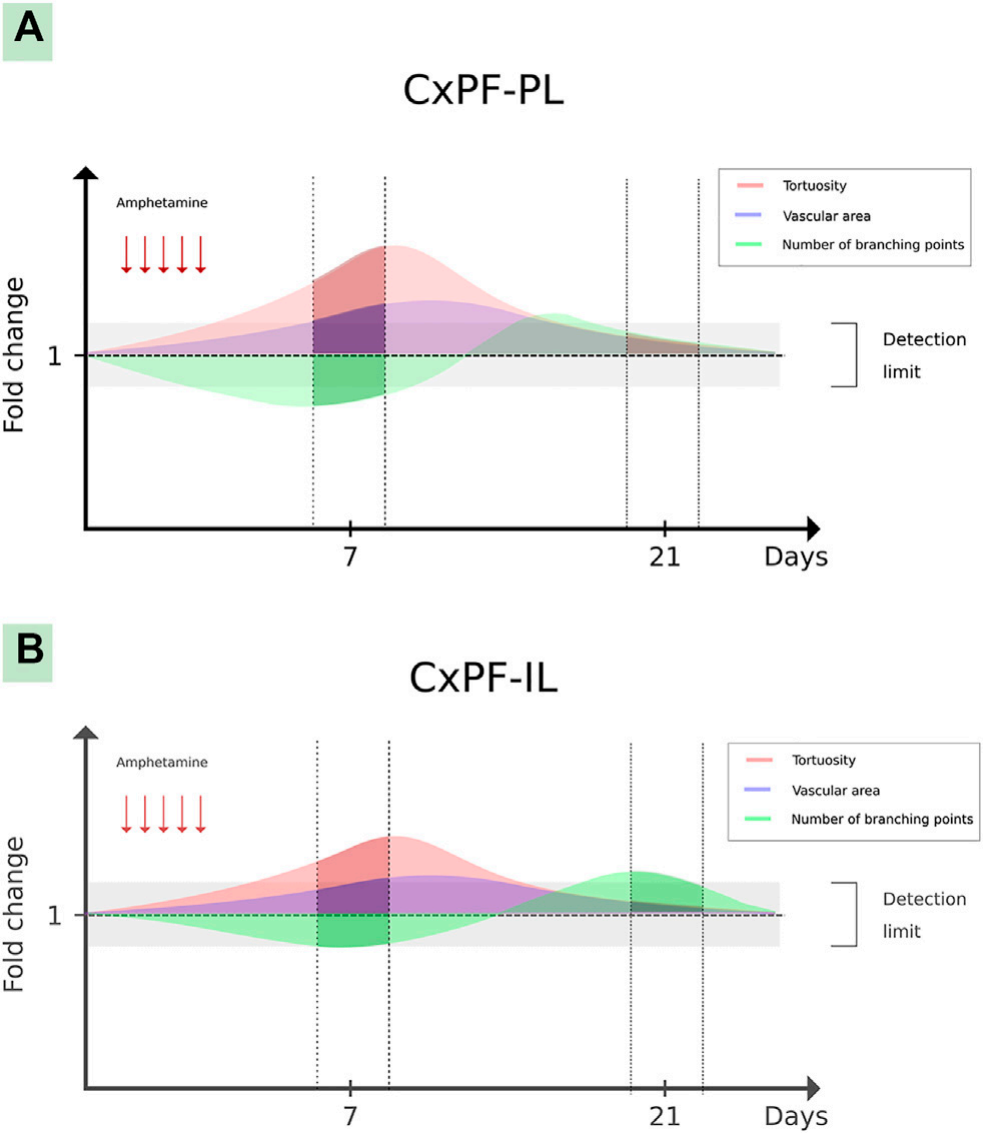
Hypothetical transient angiogenesis induced by amphetamine in the prefrontal cortex. The graph shows hypothetical angiogenic changes induced by amphetamine in the PL-PFC **(A)** and IL-PFC **(B)** subdivisions. The darker lines show the described vascular microarchitecture changes in both brain structures (in days 7 and 21 after the last amphetamine exposure), meanwhile the translucent lines show hypothetical changes in the gaps among these periods.

The extensive AMPH-induced oxidative stress via AT_1_-R, observed in the present work, may play an important role in angiogenic signaling because ROS are potential inducers of HIF-1 up-regulation. It has been found that ROS induces HIF-1 stabilization via prolyl-hydroxylase inactivation and HIF-1 transcription and transactivation (Belaidi et al., 2016; Kirtonia et al., 2020). In IL-PFC, AMPH induced an increased vascular tortuosity, via AT_1_-R, together with no changes in branching points at day 7 of withdrawal. In line with these results, in a previous work we observed an AMPH-induced increase of vascular tortuosity in PL-PFC together with less branching points and an increased microvessels occupied area (Marchese et al., 2020). On the contrary, in the present work at day 21 of withdrawal, AMPH induced an increased number of branching points together with no changes in vascular tortuosity and microvessels occupied area (data not shown) in IL-PFC, whereas tortuosity, branching and microvessels occupied area were normalized in PL-PFC. From the above and taking into account that tortuosity is considered as an early step of neovascularization (Scott et al., 2014), we hypothesize that AMPH induces angiogenesis in both subdivisions of PFC but with a different time course and magnitude. As microvascular parameters were evaluated only in two time-points (7 and 21 after the last AMPH exposure), it is important to highlight that a more detailed analysis is needed to fully describe the time-course of angiogenic changes (onset, peak and disappearance) triggered by AMPH. The hypothetical transient angiogenesis described is schematized in Figure 7.

Taking into account that in the present work CAND was orally administered and the evidence showing that it crosses the blood-brain barrier (Michel et al., 2013), the CAND prevention of the AMPH alterations could result from central or peripheral AT_1_-R antagonism or a combination of both effects.

Although, the present results show the AT_1_-R involvement in the AMPH-induced working memory deficit, oxidative stress, gliosis and angiogenesis; the prevention effectiveness of these alterations by the AT_1_-R blockade could depend on several factors such as dose, dosing regimen and extension of AMPH exposure. Therefore, studies with higher AMPH doses, different dosing regimens or with prolonged exposure to the psychostimulant would be useful to extend further the knowledge about AT_1_-R role in the AMPH-induced alterations.

## Conclusion

Overall, our results support a protective role of AT_1_-R blockade in AMPH-induced oxidative stress and the subsequent long-lasting glial activation and transient angiogenesis, preserving working memory performance. CAND and several AT_1_-R antagonists are currently used for hypertension treatment, with a low frequency of side effects, and they do not alter blood pressure in normotensive patients. Although more studies are necessary to characterize further the effectiveness of AT_1_-R blockers in DA-imbalance-related pathologies.

## Data Availability

The authors confirm that all data underlying the findings will be fully available at https://rdu.unc.edu.ar/ (Repositorio Digital de la Universidad Nacional de Córdoba).
